# Dietary Supplementation with Goji Berries (*Lycium barbarum)* Modulates the Microbiota of Digestive Tract and Caecal Metabolites in Rabbits

**DOI:** 10.3390/ani12010121

**Published:** 2022-01-05

**Authors:** Paola Cremonesi, Giulio Curone, Filippo Biscarini, Elisa Cotozzolo, Laura Menchetti, Federica Riva, Maria Laura Marongiu, Bianca Castiglioni, Olimpia Barbato, Albana Munga, Marta Castrica, Daniele Vigo, Majlind Sulce, Alda Quattrone, Stella Agradi, Gabriele Brecchia

**Affiliations:** 1 Institute of Agricultural Biology and Biotechnology (IBBA), National Research Council (CNR), U.O.S. di Lodi, Via Einstein, 26900 Lodi, Italy; cremonesi@ibba.cnr.it (P.C.); biscarini@ibba.cnr.it (F.B.); castiglioni@ibba.cnr.it (B.C.); 2 Department of Veterinary Medicine, University of Milano, Via dell’Università 6, 26900 Lodi, Italy; giulio.curone@unimi.it (G.C.); federica.riva@unimi.it (F.R.); daniele.vigo@unimi.it (D.V.); gabriele.brecchia@unimi.it (G.B.); 3 Department of Agricultural, Food and Environmental Sciences, University of Perugia, Borgo XX Giugno 74, 06121 Perugia, Italy; elisa.cotozzolo@studenti.unipg.it; 4 Department of Agricultural and Food Sciences, University of Bologna, Viale G. Fanin 44, 40137 Bologna, Italy; 5 Department of Veterinary Medicine, University of Sassari, Via Vienna, 2, 07100 Sassari, Italy; marongiu@uniss.it; 6 Department of Veterinary Medicine, University of Perugia, Via San Costanzo 4, 06126 Perugia, Italy; olimpia.barbato@unipg.it (O.B.); alda.quattrone@hotmail.it (A.Q.); 7 Faculty of Veterinary Medicine, Agricultural University of Tirana, Kodër-Kamëz, 1029 Tirana, Albania; amunga@ubt.edu.al (A.M.); msulce@ubt.edu.al (M.S.); 8 Department of Health, Animal Science and Food Safety “Carlo Cantoni”, University of Milan, Via dell’Università 6, 26900 Lodi, Italy; marta.castrica@unimi.it

**Keywords:** Goji fruit, intestinal bacterial community, caecum, lactic acid, ammonium, rabbit

## Abstract

**Simple Summary:**

The microbial community that inhabits specific areas of the body, developing a symbiotic relationship with the host, is termed the microbiota. The intestinal microbiota plays a pivotal role in different physiological processes and is influenced by many factors, including nutrition. Goji berries are a popular nutraceutical product that have been proposed as a dietary supplement in some livestock species, including rabbits, but their effects on the composition of the microbiota have never been investigated. This study evaluated the effects of Goji berry supplementation on the microbiota of different digestive tracts (stomach, duodenum, jejunum, ileum, caecum and colon) of the rabbit, using a modern method of analysis. Our results suggest that Goji berries could modulate the microbiota of the rabbit’s digestive tract increasing the growth of beneficial bacteria, such as Ruminococcaceae, Lachnospiraceae, Lactobacillaceae, and particularly, the genus *Lactobacillus*. These findings suggest that Goji berries could be used to produce innovative feeds for rabbits, although further studies are necessary to evaluate their impact on productive performance, gut immune system maturation, as well as resistance to gastrointestinal disorders.

**Abstract:**

Goji berries show health benefits, although the possible mechanisms of action, including compositional changes in the gut microbiome, are still not fully understood. The aim of this study was to evaluate the effect of Goji berry supplementation on microbiota composition and metabolites in the digestive tracts of rabbits. Twenty-eight New Zealand White rabbits were fed with a commercial feed (control group, C; *n* = 14) or the same diet supplemented with 3% of Goji berries (Goji group, G; *n* = 14), from weaning (35 days old) until slaughter (90 days old). At slaughter, samples from the content of the gastrointestinal tracts were collected and analyzed by Next Generation 16S rRNA Gene Sequencing to evaluate the microbial composition. Ammonia and lactic acid were also quantified in caecum. Results showed differences in microbiota composition between the groups for two phyla (Cyanobacteria and Euryarchaeota), two classes (Methanobacteria and Bacilli), five orders, fourteen families, and forty-five genera. Ruminococcaceae (*p* < 0.05) and Lachnospiraceae (*p* < 0.01) were more abundant in G than in C group. Lactobacillaceae also showed differences between the two groups, with *Lactobacillus* as the predominant genus (*p* = 0.002). Finally, Goji berry supplementation stimulated lactic acid fermentation (*p* < 0.05). Thus, Goji berry supplementation could modulate gastrointestinal microbiota composition and caecal fermentation.

## 1. Introduction

Goji berries, the fruits of the *Lycium barbarum* plant, are often used in traditional Chinese medicine for their nutritional and therapeutic properties, and are also widespread as supplementation in Western diets [1,2]. Their health benefits are associated with biologically active compounds, including polysaccharides, carotenoids, polyphenols, amino acids, ascorbic acid, and unsaturated fatty acids [3], although their mechanisms of action are still not fully understood. Recent evidence has shown that the fruit could modulate the gut microbiota and thus have a role in the prevention and treatment of several gastrointestinal diseases in mice [4,5], rats [6] and humans [7]. Recently, Goji berries have also been proposed as a dietary supplement for some livestock species, with the dual aim of improving productive performance and product quality [8,9,10]. In rabbits, Goji berry supplementation seems to improve reproductive [11] and productive performances, [12] energy metabolism [13], and meat quality [14,15] in a dose-dependent manner, but its effects on gut microbiota have not yet been investigated.

The microbiota represents a complex ecosystem of microorganisms which inhabits specific niches of the body and plays important roles in physiological processes developing symbiotic relationships with the host [16]. The intestinal microbiota is involved in the digestion and absorption of nutrients, maturation and stimulation of the immune system, as well as protection against pathogenic infections [17]. The bacterial microbiota composition along the gastrointestinal tract of adult rabbits fed with a commercial diet has recently been characterized [18]. This study showed interesting differences among the various sections of the digestive system in bacterial richness and diversity [18]. Within the same species, however, bacterial community composition of the gastrointestinal tract can be influenced by several factors, including nutrition [13]. Goji supplementation could therefore induce favorable changes in the intestinal microbiota of the rabbit with beneficial effects on health and productive performance, as seen in other animal species and humans [4,5,6,7,8,9,10].

The rabbit is a very interesting species because it can be a pet, livestock or animal model. Both in pet and farmed rabbits, the digestive system is a common site of diseases that are often associated with changes in intestinal microbiota [19,20]. In particular, the peri-weaning period is the most critical physiological phase as the diet transition induces changes in the gut microbiota increasing sensitivity to gastrointestinal pathogens [21]. Antibiotics are commonly used to control intestinal infections; however, according to recommendations of the European Union, this practice should be reduced [22,23]. An innovative strategy to limit the incidence of gastrointestinal disorders could be the use of specific feeds for pet and farmed rabbits integrated with nutraceutical products such, as *Lycium barbarum* fruit, to favor the growth of a beneficial gut microbiota. Understanding the effects of Goji berry supplementation on the intestinal microbiota can therefore have important implications for the health of rabbits. The rabbit could be also considered as an animal model for diet-induced changes in gut microbiota, as it has already been used for studies exploring the effect of nutrition on productive [24,25,26], reproductive [27,28], and immunological traits [29,30].

The aim of this study was to investigate the effect of Goji berry supplementation on the microbiota composition of the different tracts of the digestive system (stomach, duodenum, jejunum, ileum, caecum, and colon) in the rabbit. For this purpose, the microbiota of all the sections of the digestive apparatus was analyzed using Next Generation 16S rRNA Gene Sequencing. In addition, metabolites from bacterial fermentation in the caecum (lactic acid and ammonia) were also quantified.

## 2. Materials and Methods

### 2.1. Animals and Samples Collection

The experimental trial was conducted in the facilities of the Faculty of Veterinary Medicine of the Agricultural University of Tirana, Tirana, Albania.

The rabbits were maintained under the supervision of a responsible veterinarian and in accordance with the Directive 2010/63/EU regarding the protection of animals kept for farming purposes. The lowest number of rabbits necessary to obtain reliable results was used for the trial.

According to dietary treatment, 28 New Zealand White male rabbits were randomly assigned into two groups from weaning (35 days of age) until slaughter (90 days of age): control group (*n* = 14 animals, C), fed with a commercial pellet, and Goji group (*n* = 14 animals, G), fed with the same feed of the C group supplemented with 3% of Goji berries (Gianluca Bazzica, Foligno, Italy) before pelleting (Table 1). At weaning the average body weight was 875 ± 115 g and 893 ± 135 in C and G groups, respectively. Feeds from the same batches were previously used in other experiments [11,13,14,15].

Rabbits were bred in single cages and maintained at a temperature range between 18 and 21 °C, relative humidity of 60%, and with a photoperiod of 16 h of light. Throughout the entire trial, water and feed were provided ad libitum.

At the slaughterhouse, the gastrointestinal tract was immediately removed from each rabbit. The content of the different digestive tract sections from each animal (stomach, duodenum, jejunum, ileum, caecum, and colon) were collected separately in 15 mL sterile tubes and then stored at −80 °C until examination. Each sample was analyzed individually. The average body weights (±standard error) at weaning were 875 ± 55 g and 893 ± 75, while at slaughter, they were 2310 ± 82 g and 2357 ± 82 g in C and G groups, respectively.

### 2.2. Microbiota Evaluation—Genomic Sequencing

#### 2.2.1. DNA Extraction

Using the commercial QIAamp PowerFecal Pro DNA Kit (Qiagen, Hilden, Germany), the bacterial DNA was extracted from each sample of intestinal contents following the manufacturer’s protocol. DNA quality and quantity were checked using a NanoDrop ND-1000 spectrophotometer (NanoDrop Technologies, Wilmington, DE, USA, and the obtained DNA was stoked at −20 °C until use.

#### 2.2.2. 16S Ribosomal RNA (rRNA) Gene Sequencing

Bacterial DNA was amplified using primers described in the literature [32] which target the V3-V4 hypervariable regions of the 16S rRNA gene. All the PCR amplifications were performed in 25 µL volumes per sample. A total of 12.5 µL of KAPA HIFI Master Mix 2× (Kapa Biosystems, Inc., Wilmington, MA, USA) and 0.2 µL of each primer (100 µM) were added to 2 µL of genomic DNA (5 ng/µL). Blank controls (no DNA template added to the reaction) were also performed. A first amplification step was performed in an Applied Biosystem 2700 thermal cycler (ThermoFisher Scientific, Waltham, MA, USA). The samples were denatured at 95 °C for 3 min, followed by 25 cycles with a denaturing step at 98 °C for 30 s, annealing at 56 °C for 1 min, and extension at 72 °C for 1 min, with a final extension at 72 °C for 7 min. The amplicons were then cleaned with Agencourt AMPure XP (Beckman, Coulter Brea, CA, USA), and libraries were prepared following the 16S Metagenomic Sequencing Library Preparation Protocol (Illumina, San Diego, CA, USA). The libraries obtained were quantified using Real Time PCR with KAPA Library Quantification Kits (Kapa Biosystems, Inc., Wilmington, MA, USA), pooled in equimolar proportion, and then sequenced in one MiSeq (Illumina, San Diego, CA, USA) run with 2 × 250-base paired-end reads.

#### 2.2.3. Sequence Analysis

The reads obtained by the 16S rRNA sequencing were analyzed as previously described [18]. One rabbit from the Goji group and two samples, both from G diet (caecum intestinal tract), were removed because they had a total number of counts <100.

#### 2.2.4. Alpha and Beta Diversity Indices

To assess the microbial diversity of the different rabbit gastrointestinal tracts the alpha (within-) and beta (across-) diversities were used. These indices were estimated starting from the OTU table, after filtering with more than 50 total counts, distributed in at least five samples. Besides the number of observed OTUs directly, within-sample microbial richness, diversity, and evenness were estimated using Chao1 and ACE (abundance-based coverage estimator) for richness, Shannon, Simpson, and Fisher’s alpha for diversity [33,34], and Simpson E and Pielou’s J (Shannon’s evenness) for evenness [35]. The Bray–Curtis dissimilarity [36] was used to quantify the across-sample microbiota diversity. Prior to the calculation of these metrics, the OTU counts were normalized for uneven sequencing depth by cumulative sum scaling (CSS) [37]. Details of these analyses can be found in Biscarini et al. [38].

#### 2.2.5. Software

The QIIME 1.9 pipeline [39] was utilized both to analyze the reads obtained from 16S rRNA gene sequencing and to estimate most diversity indices. Own Python (https://github.com/filippob/Rare-OTUs-ACE.git, accessed on 15 November 2021) and R (https://github.com/filippob/sampleBasedRarefaction, accessed on 15 November 2021) scripts were used to estimate the ACE index and sample-based rarefaction. The figures were generated with the ggplot2 R package [40]. The R environment for statistical computing [41] was used to perform the additional data handling and statistical analysis.

### 2.3. Lactic Acid and Ammonia Quantification

For the analysis of bacterial metabolites (lactic acid and ammonia), 1 g of caecal content was diluted in 1 mL of 1 M perchloric acid and 8 mL of distilled water. After homogenization, tubes were centrifuged for 10 min at 5000 rpm, and the supernatant was transferred to 2 mL Eppendorf tube and frozen at −20 °C until metabolite quantification. The spectrophotometric method for biological fluids was used for lactic acid determination in accordance with Pryce et al. [42]. Ammonia concentration was detected in line with Patton et al. [43]. Spectrophotometer was set at 565 nm and 660 nm respectively (Shimadzu Corporation UV-2550, Kyoto, Japan). All chemicals were purchased from Sigma Chemical Co (St. Louis, MO, USA).

### 2.4. Statistical Analysis

Differences in alpha diversity indexes between treatments at various taxonomic levels along the rabbit’s gastrointestinal tract were tested with a linear model that took into account the hierarchical structure of within-subject nested data (consecutive sections of the gastrointestinal tract belonging to individual rabbits). The model had the following form:

y_ijkt_ = µ + rabbit_j_ + treatment_k_ + anatomic region_t(j)_ + e_ijkt_ (1)

where y_ijkt_ is the alpha diversity index value for record i from rabbit j with treatment k and anatomic region t, µ is the intercept, rabbit_j_ is the systematic effect of the individual rabbits, treatment_k_ is the treatment effect (Goji vs. control), anatomic region_tk(j)_ is the effect of the anatomic region of the gastrointestinal tract nested within rabbit_j_, and e_ijkt_ is the residual.

Var(y) = Sigma + Iσ_e_^2^

where Sigma is a block diagonal matrix, with 1 s on the diagonal and the covariances σ_ij_ between records within rabbits in the off-diagonal block elements, I is the identity matrix, and σ_e_^2^ is the residual variance.

A simplified version of Model (1) was used to evaluate differences between Goji and control samples; in particular, where the anatomic region effect was dropped and data from all gastrointestinal sections were analyzed jointly to evaluate the effect of Goji supplementation on the overall rabbit gut microbiota.

For Bray–Curtis dissimilarities (beta diversity), differences along the digestive tract were tested non-parametrically using the permutational analysis of variance approach (999 permutations; [33]).

## 3. Results

### 3.1. Sequencing Results

The microbiota structure of the gastrointestinal tract of C and G groups was characterized by a total of 6,122,359 and 7,156,769 high quality reads (after filtering), respectively, with a mean of 75,584 ± 38,864 reads for C and 90,592 ± 33,296 reads for G group. The evaluation of the sample-based and sequence-based rarefaction curves suggested that the depth of coverage was sufficient to describe the biological diversity within the samples (Figure S1).

### 3.2. Taxonomic Composition of Gut Microbiota along the Rabbit Gastrointestinal Tract of C and G Groups

**Figure 1:**
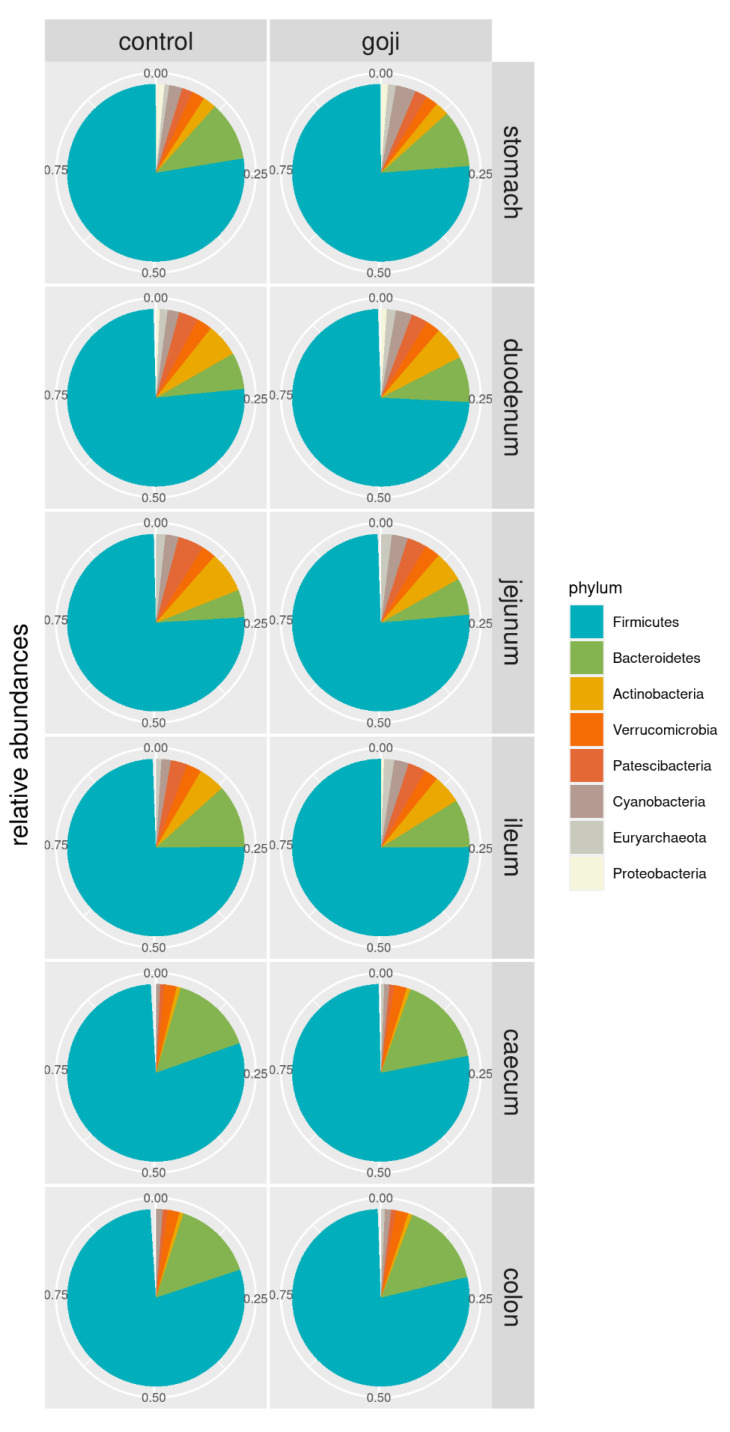
Pie-chart of phylum relative abundances in control and Goji-treated rabbits along the gastrointestinal tract. For the analyses, 14 and 13 samples were used for the control and Goji groups, respectively.

Phylum relative abundances distribution along the gastrointestinal tract of C and G groups are summarized in Figure 1. Significative differences were found in microbiota composition between the experimental groups for two phyla, two classes, five orders, fourteen families, and forty-five genera (Table S1). Firmicutes represented the main phylum in all sections of the digestive tract, especially in the most distal portions of caecum and colon (77–79% of total bacteria) for both groups, and Bacteroidetes the second (14–16% of total bacteria). The caecum and colon of rabbits treated with Goji berries showed differences regarding the abundance of Bacteroidetes (16%) compared to the control group (14%) although these were not statistically significant. As regards other phyla, Actinobacteria was present in the upper part of the gastrointestinal tract. In the jejunum, its relative abundance was higher in C than G group (7.5% for C vs. 5.5% for G group), while in the ileum the percentages were opposite (4.8% for C vs. 5.2% for G group); as with the Bacteroidetes, the differences regarding Actinobacteria were not significant. On the other hand, at the phylum level, Cyanobacteria and Euryarchaeota, the latter belonging to kingdom Archaea, were statistically different (*p* = 0.034 and *p* = 0.004, respectively) between the experimental groups, with higher relative abundances in the upper part of the gastrointestinal tract in G group.

**Figure 2:**
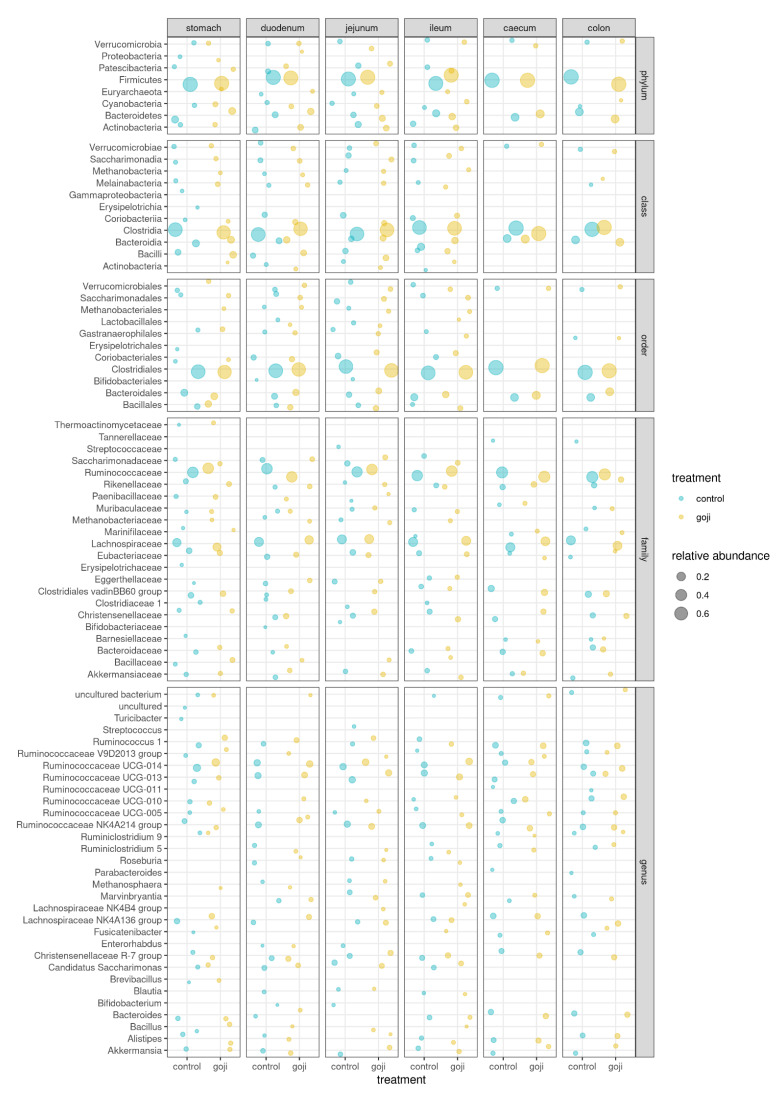
Bubble chart of relative abundances of all taxa (≥1%) in the microbiota of the digestive tract of rabbits, grouped by taxonomic level. Control (blue = 14 rabbits) and Goji (yellow = 13 rabbits) experimental groups. The size of the bubble is proportional to the relative abundance, with 0.2, 0.4 and 0.6 hallmarks, as shown in the legend.

Moreover, Clostridia represented the major class in all anatomic regions, while Ruminococcaceae and Lachnospiraceae were the most abundant families in the Goji group (Figure 2).

**Figure 3:**
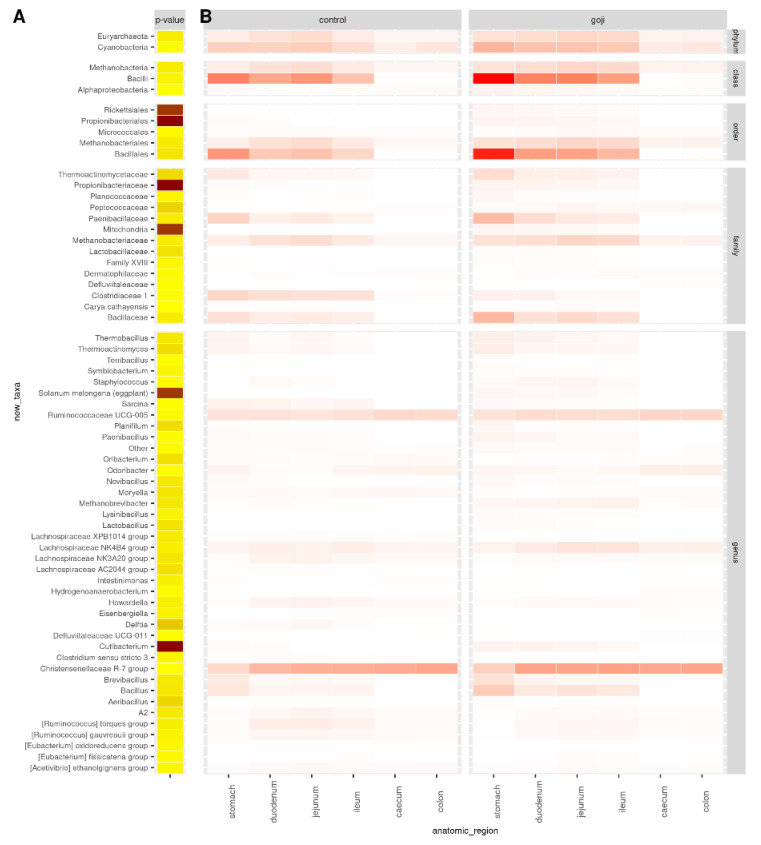
Significantly different OTUs. OTU significantly different between treatments from analysis of variance based on normalized counts: *p*-values (**A**) and counts per group and anatomic region of the rabbit digestive tract (**B**). *p*-value < 0.05 was used as cut-off. Darker colours indicate lower *p*-values (**A**) or higher counts (**B**). *p*-values are in the range 10^−15^–0.049, from dark brown to light yellow. For the analyses, 14 and 13 samples were used for the control and Goji groups, respectively.

Figure 3 shows the comparison of the relative abundances of significant OTUs between treatments and along the rabbit’s gastrointestinal tract. As shown in Table S1 and Figure 3, there were significant differences between the groups; Bacillales were predominant (*p* = 0.0032) in the G group, and *Bacillus* was the major genus in the stomach (*p* = 0.0036). Ruminococcaceae UCG-005, Lachnospiraceae NK4B4 group, and Christensenellaceae R-7 group were genera detected in all the digestive tracts with statistically significant different results between the groups. As reported in Table S1, the Lactobacillaceae family was significantly different (*p* = 0.0018) between the groups with *Lactobacillus* as the predominant genus in G group compared to C group.

### 3.3. F/B Ratio

**Figure 4:**
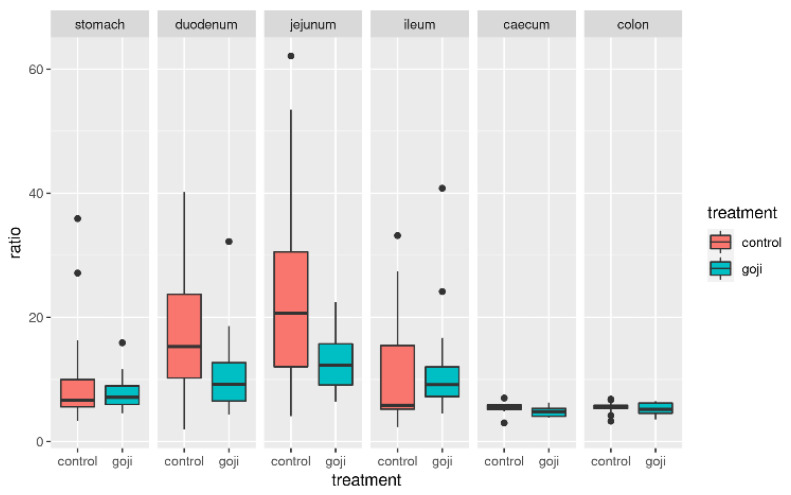
Distribution of the F:B ratio (Firmicutes to Bacteroidetes) in control and Goji-treated groups along the gastrointestinal tract. For the analyses, 14 and 13 samples were used for the control and Goji groups, respectively.

**Figure 5:**
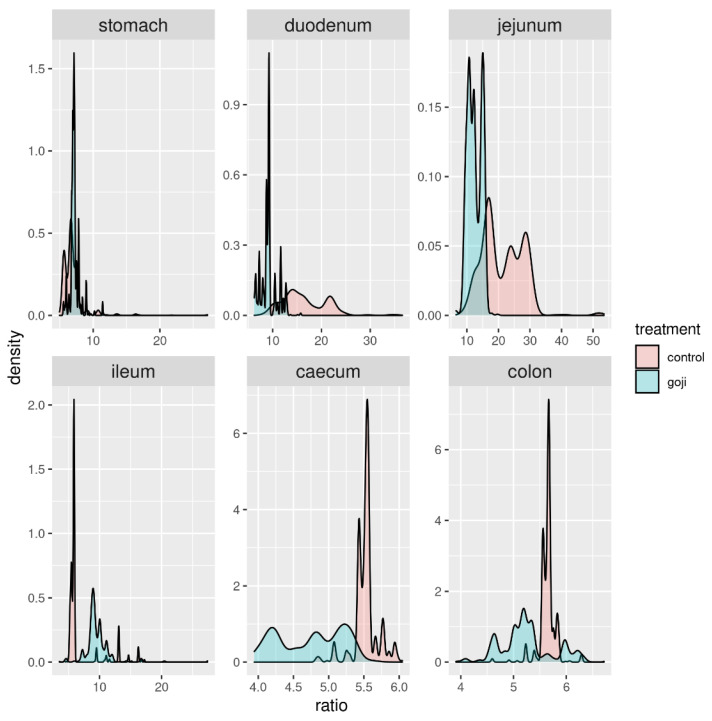
Distribution of the F:B ratio (x-axis) along the digestive tract in Goji-treated (blue) and control (red) rabbits from 1000 bootstrapping replicates of the data. For the analyses, 14 and 13 samples were used for the control and Goji groups, respectively.

The Firmicutes: Bacteroidetes (F:B) ratio followed a clear pattern along the rabbit’s digestive tract starting at around 10 in the stomach, increasing clearly in the duodenum and jejunum, and finally decreasing again in the caecum and colon. The F:B ratio appeared to be significantly lower in the G group (Figure 4), in the duodenum (*p* = 0.0176) and jejunum (*p* = 0.000049). This was confirmed by bootstrapping (1000 replicates resampled with replacement from the original data, Figure 5), which provided further statistical support of the significance of F:B differences between G and C groups in the duodenum, jejunum and, slightly less so, in the ileum.

### 3.4. Alpha Diversity Index—Treatment by Region

Table 2 reports the values for the alpha diversity indexes estimated in the rabbits’ gastrointestinal tract, in the two groups. Alpha diversity indexes were significantly different between treatments in the last portion of the digestive tract (Figure S2): six indexes were significantly different in the jejunum (ACE, Fisher’s alpha, observed n. of OTUs, Shannon and Simpson diversity), two in the ileum (Equitability and Simpson E), three in the caecum (Chao1, ACE, Fisher’s alpha), and two in the colon (Equitability and Simpson E).

### 3.5. Beta Diversity Index (Clustering Treatment X Anatomic Portion)

**Figure 6:**
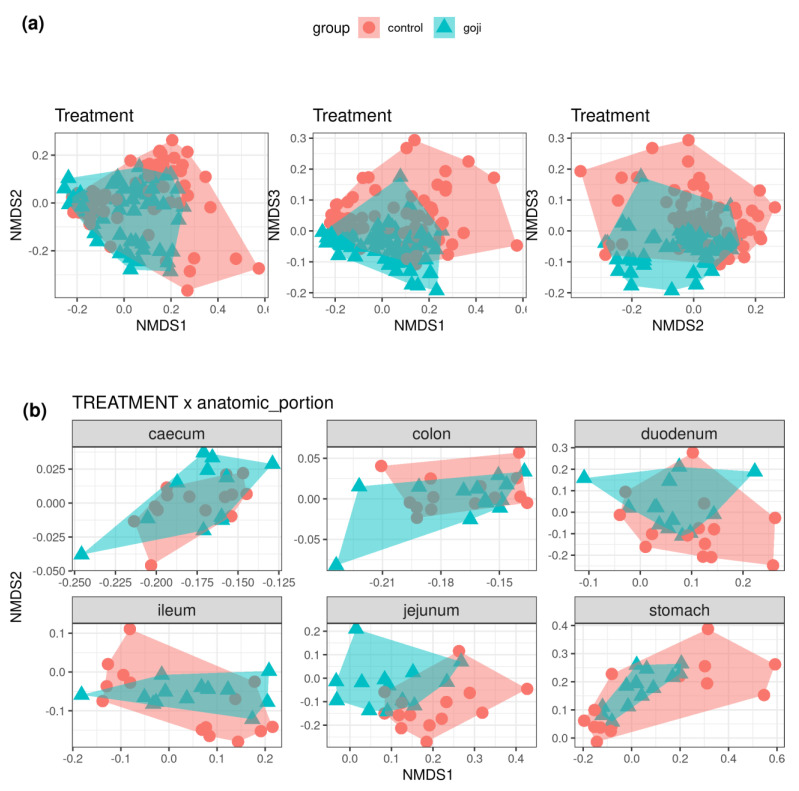
(**a**): Non-metric multidimensional scaling plot of Bray-Curtis dissimilarities estimated from the OTU table. The plots show the first three NMDS dimensions (from left to right: dimensions one and two, one and three, two and three). Control samples in red circles, Goji-treated samples in blue triangles. (**b**): First two dimensions from the non-metric dimensional scaling of Bray-Curtis dissimilarities between control and Goji-treated samples along the digestive tract of rabbits. For the analyses, 14 and 11–13 samples were used for the control and Goji groups, respectively.

Figure 6a shows the clustering of samples (C and G groups) from Bray–Curtis dissimilarities (first three dimensions from non-metric multidimensional scaling). The distance between groups were significantly different (*p* < 0.01) from permutational multivariate analysis of variance (PERMANOVA, 999 permutations). This difference appeared to vary along the gastrointestinal tract, with jejunum, caecum, and colon showing the clearest differences, while the two groups mostly overlapped in the stomach, duodenum, and ileum (Figure 6b: first two NMDS dimensions only).

### 3.6. Caecal Lactic Acid and Ammonia Quantification

Regarding lactic acid quantification, G group showed a higher concentration than C group, suggesting higher bacterial activity (3.91 ± 1.59 and 1.01 ± 1.22 mmol/kg in C and G groups, respectively; *p* = 0.033). No significant differences in ammonia concentration were detected between the two groups (5.81 ± 2.22 and 5.89 ± 1.81 mmol/kg in C and G groups, respectively; *p* = 0.305).

## 4. Discussion

Diet is one of the main factors affecting the composition of the microbiota in the digestive tract due to the relation between nutrients and microbial populations [44]. The bacterial populations inhabiting the different gastrointestinal compartments of the rabbit have been previously described [18]. For the first time, this study investigated the effect of Goji berry supplementation on microbiota composition in the different tracts of the digestive system and on caecal bacterial fermentations of adult rabbits.

The results of the present study showed a prevalence of Firmicutes in all the anatomic tracts in both experimental groups. This phylum is classified as the most efficient cellulose degrader [45] and it plays a fundamental role in rabbit digestion. Similar results were reported by both Cotozzolo et al. [18] and Arazzuria et al. [46]. This result was also supported by other studies investigating not only the caecal microbiota of rabbits [47,48] but also the gastrointestinal content and feces of both wild and domestic rabbits [49]. This is a common condition not only in hindgut fermenters, such as rabbits, but also in ruminants and monogastric animals [50].

Bacteroidetes was the second most abundant phylum, especially in the large intestine (caecum and colon tracts), and was slightly predominant in the G group. This phylum, not significantly different between the two groups and along the digestive tracts, is known for its role in the stimulation of gut-associated lymphoid tissue [46,48]. The abundance of Bacteroidetes is in accordance with what was already observed by Cotozzolo et al. [18] on the rabbit gastrointestinal microbiota and by Crowley et al. [49] on both domestic and wild rabbits. A further analysis of our samples with a shotgun metagenomic or metatranscriptomic approach, combined with immunological assays, could provide more information about the role of this relevant phylum in gut immunity.

Regarding other phyla, Verrucomicrobia were found in all sections, while Actinobacteria and Proteobacteria were found in the stomach and small intestine. Although with low levels in the core microbiome, the Euryarchaeota phylum, belonging to the kingdom Archea, was statistically different between the two groups, with higher levels in the G group in all the digestive tracts. All species of this phylum were taxonomically assigned to the methanogenic genus *Methanobrevibacter* [51]. Though this phylum is not very common in the intestinal microflora of some species, such as horses and pigs [18], it is often found in the human gut with the role of increasing polysaccharide digestion by consuming the end products of bacterial fermentation [52].

Clostridia, anaerobic Gram-positive bacteria present in the intestinal microbiota of human, mouse, chicken, and pig, represented the major class in all anatomic regions, in accordance with Velasco-Galilea and co-workers [51]; they are prevalent, cellulose-degrading symbiotic microorganisms, helping the rabbit for plant material digestion [51].

The families of Ruminococcaceae and Lachnospiraceae were present in all anatomic parts, and both were higher in the G group. Ruminococcaceae are usually prevalent in healthy rabbits [53], while Lachnospiraceae is known to be associated with a decrease of mortality [54]. These two families appear to have an important role in fiber digestion, in particular of peptose and cellulose [55], and are significant producers of short-chain fatty acids [56]. Moreover, as previously reported [4], in mice a diet with Goji supplementation promotes butyrate-producing bacteria, including Lachnospiraceae and Ruminococcaceae families, preventing colitis; their high levels in the digestive apparatus also allow protective and beneficial effects towards different diseases, such as diabetes and heart disease [57].

Lactobacillaceae was another family that showed significant differences between the two groups, although present in small quantities. Within this family, *Lactobacillus* was the predominant genus. Lactobacilli are rare in the rabbit intestine, occupying less than 1% of the total intestinal bacteria [58], and their function in gut health is not fully understood. A recent study has shown that the total intestinal bacteria from rabbits tends to induce a higher inflammatory level than the total intestinal bacteria from chickens or pigs [59], probably because of the low abundance of Lactobacilli in the rabbit’s intestine. Thus, the higher *Lactobacillus* abundance in rabbits supplemented with Goji could play a protective role against inflammatory diseases. Components of commensal bacteria can alleviate intestinal inflammation by regulating the expression of both pro-inflammatory and anti-inflammatory factors. Kawashima et al. [60] reported that bacterial double-stranded RNA, abundant in *Lactobacillus bacteria*, showed a regulatory function by triggering anti-inflammatory factor IFN-β production and inhibiting pro-inflammatory factors production.

The F:B ratio was at around 10 in the stomach, then increased in the duodenum and jejunum, and subsequently progressively decreased from the ileum to the caecum and colon. The F:B ratio appeared to be lower in the G group, significantly so in the duodenum and jejunum, less so in the ileum, caecum and colon, as also shown by the bootstrapping analysis. Studies in human microbiota and in animal models, have reported that the F:B ratio was directly related to body weight modifications and in particular to obesity [61]. In obese people, the population of Firmicutes shows an elevated proportion with a reduced Bacteroidetes population; this unbalance causes an altered F:B ratio [62]. Additionally, a strong correlation between the F:B ratio and milk fat yield has been observed in dairy cattle [63]. In previous studies, feed supplementation in livestock has been reported to alter the F:B ratio in the gut microbiota (e.g., grape pomace supplementation in cattle [38]). Further studies could investigate the link between Goji intake, F:B ratio and lipid metabolism in rabbits.

The alpha diversity results revealed higher microbial richness and diversity in bacterial composition independently from the treatments in the large intestine. That was an expected result because, as already demonstrated in other livestock species, the microbial densities (and also diversity) along the gastrointestinal tract is maximal in the fermenting compartments [64]. Indeed, Cotozzolo et al. found alpha diversity of the cecum and colon to be significantly higher than for the other compartments of the rabbit gastrointestinal tract [18]. As previously reported [18], this variability, typical of colon and caecum tracts, is due essentially to their physiological functions, such as fermentation of cellulose with production of volatile fatty acids (VFA) and their absorption for energy production. Goji berry supplementation caused higher microbial richness, especially in the jejunum, ileum, and colon tracts, where six indexes were significantly different in the jejunum (ACE, Fisher’s alpha, observed n. of OTUs, Shannon and Simpson diversity), two in the ileum (Equitability and Simpson E), three in the caecum (Chao1, ACE, Fisher’s alpha), and two in the colon (Equitability and Simpson E). In particular, the principal families involved in the microbial richness were Ruminococcaceae and Lachnospiraceae, as well as *Lactobacillus* spp. These conditions could guarantee greater resilience toward dysbiosis in the gut microbiota, which is necessary to maintain homeostasis and, in turn, the healthy status of the gastrointestinal system [65]. The beta diversity was greatly influenced by Goji treatment, especially in caecum and colon tracts, which play fundamental roles in the digestion of fermenter animals, such as rabbits. Conversely, less influence of this treatment was found in the stomach, duodenum, and ileum tracts.

The differences in microbiota composition are due to the environmental conditions, such as pH modifications, along the gastrointestinal tract. In adult rabbits, the principal substrates for caecal microorganisms are polysaccharides and protein. Caecal microorganisms ferment available nutrients, converting them to metabolites (e.g., short-chain VFA, ammonia, H_2_, CH_4_, CO_2_) and compounds that are incorporated into microbial cells [66]. Our results for caecal bacterial fermentations indicate that Goji berry supplementation did not influence proteolytic activity and ammonia production. On the other hand, Goji supplementation stimulated lactic acid fermentation, indicating changes in the intestinal microbiota in favor of specific bacterial populations. The caecum represents the main site of fermentative activity in the rabbit due to the presence of an abundant microbial flora [1]. Rabbits produce large amounts of VFA and lactate by fermentation of dietary carbohydrates, such as xylan and pectin, in the hindgut [67,68,69]. Lactobacilli are strong producers of lactic acid and, for this reason, can compete against pathogenic bacteria [70]. Regarding Goji berry supplementation, several authors [71,72] have confirmed the beneficial effects of this integration on the physiology and health of the gut acting on the intestinal microbiota composition of human and mice. Castrica et al. [15] reported that the incorporation of 3% *w/w* of Goji berries in the rabbit diet was able to increase the Lactobacilli population in rabbit meat.

This is a preliminary study on the effect of Goji berry supplementation on gastrointestinal microbiota of the rabbit and, although its practical implications are currently limited, it may represent a starting point for future exploratory research. Further experimental trials could be addressed to evaluate whether caecal fermentative activities (VFA production) could be affected by changes in microbial community composition. Moreover, evaluation of digestive efficiency by performing an in vivo digestibility trial could integrate the study of the microbiota composition of the rabbit. Finally, it could be interesting to evaluate the impact of microbiota modification on the maturation and activity of the immune system, as well as on resistance to infectious diseases, animal welfare and the productive performance of the rabbit.

## 5. Conclusions

The present study demonstrated that Goji berry supplementation can modulate gastrointestinal microbiota composition and caecal fermentations of the rabbit. In particular, *Lycium barbarum* fruit increased the growth of the phylum Bacteroidetes as well as of Ruminococcaceae, Lachnospiraceae, and *Lactobacillus* in the caecum and colon, and as a consequence, lactic acid production. The mechanism of absorption and integration of the bioactive molecules contained in the fruit, and their influence on the microbiota population, should be investigated to appropriately use Goji berries’ probiotic properties. The use of this natural compound needs to be further studied for its implications for both commercial performance and animal resistance to infection, as its supplementation could reduce the incidence of health problems in livestock and consequent antibiotic treatments.

## Figures and Tables

**Table 1:** Feed formulation and chemical composition (as fed) of control group and Goji group diet.

Ingredients	Unit	Diet	
		Control	Goji
Wheat bran	%	30.0	29.0
Dehydrated alfalfa meal	%	42.0	41.0
Barley	%	9.5	9.0
Sunflower meal	%	4.5	4.2
Rice bran	%	4.0	3.9
Soybean meal	%	4.0	3.9
Calcium carbonate	%	2.2	2.2
Cane molasses	%	2.0	2.0
Dicalcium phosphate	%	0.7	0.7
Vitamin-mineral premix ^1^	%	0.4	0.4
Soybean oil	%	0.4	0.4
Salt	%	0.3	0.3
Goji berries	%	-	3.0
Chemical composition			
Crude Protein	%	15.74	15.66
Ether extract	%	2.25	2.47
Ash	%	9.28	9.25
Starch	%	16.86	16.99
NDF	%	38.05	37.49
ADF	%	19.54	19.01
ADL	%	4.01	3.98
Digestible Energy ^2^	MJ/Kg	10.3	10.3

^1^ Per kg diet: vitamin A 11,000 IU; vitamin D3 2000 IU; vitamin B1 2.5 mg; vitamin B2 4 mg; vitamin B6 1.25 mg; vitamin B12 0.01 mg; alpha-tocopherol acetate 50 mg; biotine 0.06 mg; vitamin K 2.5 mg; niacin 15 mg; folic acid 0.30 mg; D-pantothenic acid 10 mg; choline 600 mg; Mn 60 mg; Fe 50 mg; Zn 15 mg; I 0.5 mg; Co 0.5 mg. ^2^ NDF: Neutral Detergent Fiber; ADF: Acid Detergent Fiber; ADL: Acid Detergent Lignin. Estimated by Maertens et al. [31].

**Table 2:** Alpha diversity indices along the digestive tract of rabbits in the two experimental groups (14 controls and 13 Goji-treated; two more samples, both from caecum intestinal G diet were removed because they had a total number of read counts < 100). * indicates significant difference (*p* < 0.05) between control and Goji groups.

Group	Anatomic Portion	N	Chao1	Ace	Fisher Alpha	Observed OTUS	Shannon	Simpson	Equitability	Simpson E
Control	Stomach	14	378.115	380.038	163.32	335.786	7.779	0.993	0.973	0.747
Goji	Stomach	13	320.39	318.117	135.593	300.923	7.926	0.995	0.976	0.766
Control	Duodenum	14	279.333	274.565	120.298	268.143	7.806	0.995	0.976	0.764
Goji	Duodenum	13	329.489	325.595	142.572	312.462	7.999	0.995	0.975	0.750
Control	Jejunum	14	205.000 *	205.000 *	85.734 *	205.000 *	7.427 *	0.993 *	0.979	0.787
Goji	Jejunum	13	306.591 *	305.284 *	130.950 *	287.000 *	7.878 *	0.995 *	0.975	0.750
Control	Ileum	14	341.365	345.910	152.660	327.286	8.034	0.995	0.975 *	0.749 *
Goji	Ileum	13	410.027	396.468	169.947	355.000	8.149	0.996	0.968 *	0.700 *
Control	Caecum	14	683.149 *	640.207 *	274.313 *	534.714	8.734	0.997	0.965	0.674
Goji	Caecum	11	553.633 *	555.73 *	235.517 *	494.909	8.642	0.997	0.966	0.687
Control	Colon	14	621.580	616.796	271.393	529.929	8.731	0.997	0.966 *	0.682 *
Goji	Colon	13	656.245	638.687	265.692	543.385	8.744	0.997	0.964 *	0.666 *

## Data Availability

The data presented in this study are available in the article and Supplementary materials. Further information is available upon request from the corresponding author.

## Supplementary Materials

The following are available online at https://www.mdpi.com/article/10.3390/ani12010121/s1: Figure S1: Evaluation of the sample-based and sequence-based bacterial rarefaction curves to check the depth of coverage; Figure S2: *p*-values for the difference in alpha diversity indices between control and Goji-treated rabbits, along the gastrointestinal tract; Table S1: Significantly different OTUs between Goji-treated and control rabbits in the gut microbiota (*p* < 0.05).
