# Supplementary material for: Dietary Supplementation with Goji Berries (Lycium barbarum) Modulates the Microbiota of Digestive Tract and Caecal Metabolites in Rabbits
Source: Animals (Basel). 2022 Jan 5;12(1):121. doi: 10.3390/ani12010121 (PMC8749899; doi:10.3390/ani12010121)
Supplement: Supplementary file 1 [file animals-12-00121-s001.zip › animals-1508948-supplementary.pdf]

Supplementary materials

Table S1. Significantly different OTUs between Goji-treated and control rabbits in the gut microbiota ($p < 0.05$). OTUs are grouped and ordered by taxonomic level.

Level	New_taxa	Term	*p*-value
Phylum	Cyanobacteria	Treatment	0.034474138
Phylum	Euryarchaeota	Treatment	0.004589084
Class	Bacilli	Treatment	0.015972341
Class	Methanobacteria	Treatment	0.004589084
Order	Bacillales	Treatment	0.003293078
Order	Methanobacteriales	Treatment	0.004589084
Order	Micrococcales	Treatment	0.021952641
Order	Propionibacteriales	Treatment	0.000000004
Order	Rickettsiales	Treatment	0.000000017
Family	Bacillaceae	Treatment	0.007467206
Family	Christensenellaceae	Treatment	0.045257535
Family	Clostridiaceae 1	Treatment	0.025859043
Family	Defluviitaleaceae	Treatment	0.022785570
Family	Dermatophilaceae	Treatment	0.027665389
Family	Family XVIII	Treatment	0.019420602
Family	Lactobacillaceae	Treatment	0.001800593
Family	Methanobacteriaceae	Treatment	0.004589084
Family	Mitochondria	Treatment	0.000000017
Family	Paenibacillaceae	Treatment	0.007016148
Family	Peptococcaceae	Treatment	0.000160420
Family	Planococcaceae	Treatment	0.016096493
Family	Propionibacteriaceae	Treatment	0.000000004
Family	Thermoactinomycetaceae	Treatment	0.001003246
Genus	[Acetivibrio] ethanolgignens group	Treatment	0.019951185
Genus	[Eubacterium] fissicatena group	Treatment	0.021816711
Genus	[Eubacterium] oxidoreducens group	Treatment	0.022836309
Genus	[Ruminococcus] gauvreauii group	Treatment	0.009420603
Genus	[Ruminococcus] torques group	Treatment	0.004995398
Genus	A2	Treatment	0.003213852
Genus	Aeribacillus	Treatment	0.000391870
Genus	Bacillus	Treatment	0.003641939
Genus	Brevibacillus	Treatment	0.006003995
Genus	Christensenellaceae R-7 group	Treatment	0.031217248
Genus	Clostridium sensu stricto 3	Treatment	0.014915748
Genus	Cutibacterium	Treatment	0.000000004
Genus	Defluviitaleaceae UCG-011	Treatment	0.022785570
Genus	Delftia	Treatment	0.000037421
Genus	dgA-11 gut group	Treatment	0.017195412
Genus	Eisenbergiella	Treatment	0.015038910
Genus	Howardella	Treatment	0.006429800
Genus	Hydrogenoanaerobacterium	Treatment	0.005349264
Genus	Intestinimonas	Treatment	0.007919486

Genus	Lachnospiraceae AC2044 group	Treatment	0.001258252
Genus	Lachnospiraceae NK3A20 group	Treatment	0.002553156
Genus	Lachnospiraceae NK4B4 group	Treatment	0.006247137
Genus	Lachnospiraceae XPB1014 group	Treatment	0.003982549
Genus	Lactobacillus	Treatment	0.001800593
Genus	Lysinibacillus	Treatment	0.014481758
Genus	Methanobrevibacter	Treatment	0.002911108
Genus	Moryella	Treatment	0.002802719
Genus	Novibacillus	Treatment	0.004077850
Genus	Odoribacter	Treatment	0.028204867
Genus	Oribacterium	Treatment	0.001299886
Genus	Other	Treatment	0.033730769
Genus	Paenibacillus	Treatment	0.037093665
Genus	Planifilum	Treatment	0.001268503
Genus	Roseburia	Treatment	0.049833659
Genus	Ruminiclostridium 1	Treatment	0.013353148
Genus	Ruminococcaceae UCG-005	Treatment	0.014962080
Genus	Ruminococcaceae UCG-007	Treatment	0.016071297
Genus	Ruminococcaceae UCG-010	Treatment	0.025302373
Genus	Sarcina	Treatment	0.033709326
Genus	Solanum melongena (eggplant)	Treatment	0.000000017
Genus	Staphylococcus	Treatment	0.030172544
Genus	Symbiobacterium	Treatment	0.019420602
Genus	Terribacillus	Treatment	0.047458506
Genus	Thermoactinomyces	Treatment	0.001940909
Genus	Thermobacillus	Treatment	0.004471494

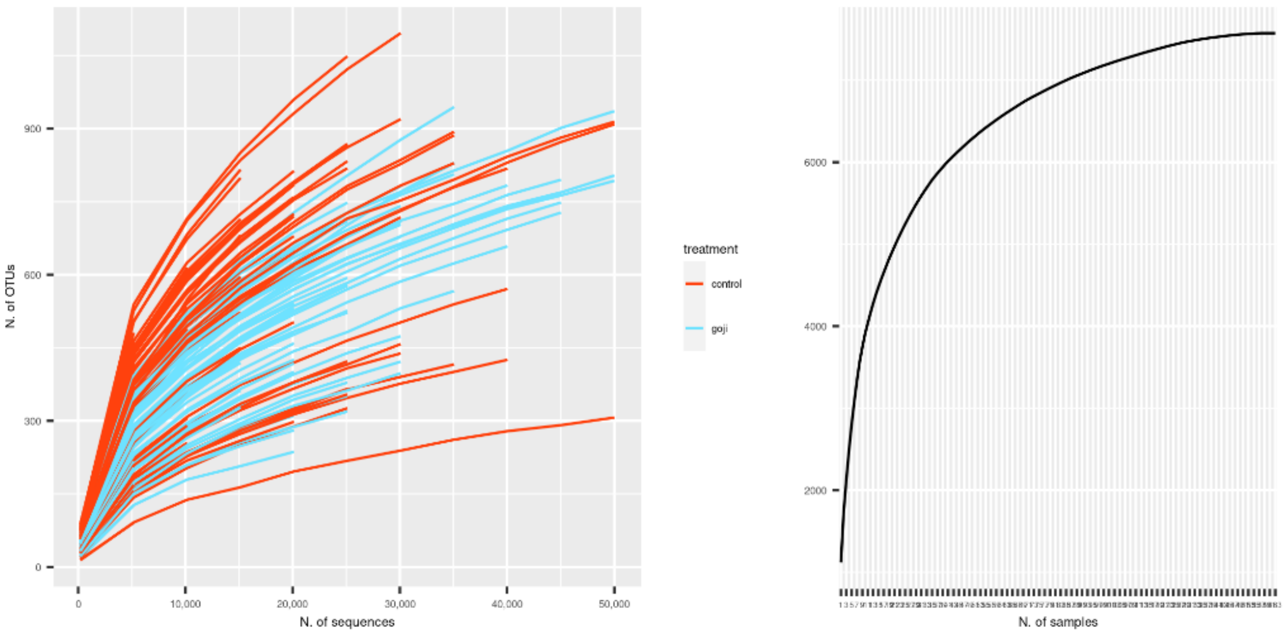

Figure S1. Evaluation of the sample-based and sequence-based bacterial rarefaction curves to check the depth of coverage.

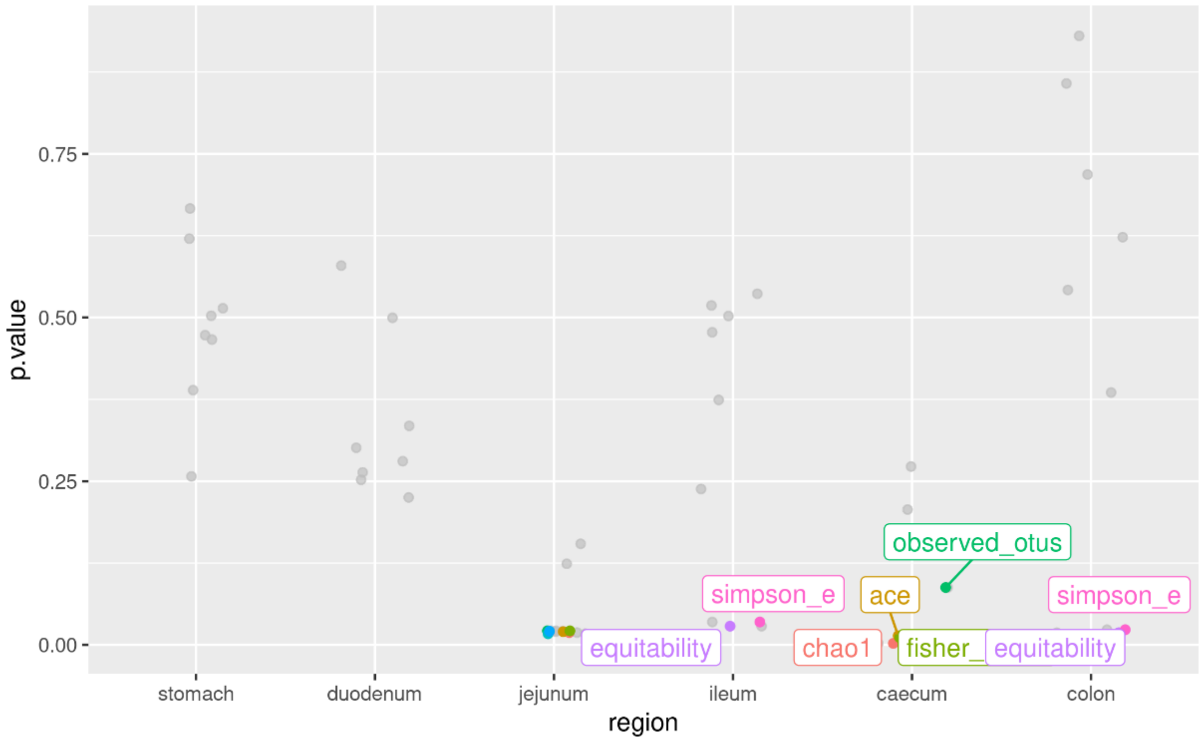

Figure S2. P-values for the difference in alpha diversity indices between control and Goji-treated rabbits, along the gastrointestinal tract. Significance was defined as $p < 0.05$. Significant differences are highlighted (colored) and labeled.
